# Aesthetic and Functional Surgical Management of Microform Cleft Lip With a Vermilion Border Incision

**DOI:** 10.7759/cureus.79337

**Published:** 2025-02-19

**Authors:** Ken Kitagawa, Hideto Imura, Masaaki Ito, Nagana Natsume, Nagato Natsume

**Affiliations:** 1 Cleft Lip and Palate Center, Aichi Gakuin University, Nagoya, JPN; 2 Division of Research and Treatment for Oral and Maxillofacial Congenital Anomalies, School of Dentistry, Aichi Gakuin University, Nagoya, JPN

**Keywords:** incomplete cleft lip, microform cleft, postoperative outcomes, surgical technique, vermilion border incision

## Abstract

A microform cleft lip is a mild form of incomplete cleft lip, often marked by small notches in the lip, a raised Cupid’s bow, and slight nasal asymmetry. Various surgical techniques, such as the rotation-advancement method and the small triangular flap method, are commonly used. However, incisions in the white lip tissue may lead to scarring or textural irregularities. This report presents the case of a 12-year-old boy with a right-sided microform cleft lip who underwent a surgical approach designed to optimize both aesthetics and function. The procedure employed an incision along the vermilion border, avoiding the white lip to preserve its natural appearance and minimize visible scarring. A triangular flap was created, and the discontinuity of the orbicularis oris muscle was corrected through overlapping and reinforcement. The intraoral incision was precisely aligned with the natural lip print, ensuring seamless integration with the surrounding tissue. Notably, the surgery was performed without local anesthetic injections, relying instead on manual compression of the labial artery for hemostasis. One year postoperatively, the patient exhibited excellent aesthetic and functional outcomes with minimal scarring. This technique underscores the importance of balancing aesthetics and function while minimizing surgical invasiveness in the management of microform cleft lip. Long-term follow-up has been planned to further assess the stability of these results.

## Introduction

A microform cleft lip is a mild form of incomplete cleft lip in which the lip tissue remains partially connected, unlike a typical cleft. While no standardized definition exists, the condition is commonly characterized by notches on the vermilion border, notches on the free edge of the vermilion, linear scars on the white lip, and mild nasal deformities [1]. Even in the absence of a visible cleft, studies have reported discontinuity in the orbicularis oris muscle [2]. This congenital anomaly is rarely documented, with an estimated incidence of approximately 0.06 per 10,000 live births [3].

The exact etiology of microform cleft lip remains unclear. However, research by Suzuki et al. [4] suggests a significant association between microform cleft lip, orbicularis oris muscle defects, and mutations in BMP4, indicating a potential genetic component in its development.

Due to its subtle clinical presentation, many individuals with microform cleft lip do not seek medical attention and may be unaware of the anomaly. Diagnosing this condition based solely on surface morphology is challenging, making advanced imaging techniques such as 3D facial measurements and ultrasonography valuable tools for detecting orbicularis oris muscle discontinuity [5]. These technological advancements have significantly improved diagnostic accuracy.

This report presents a case of microform cleft lip surgically treated with a minimally invasive approach, achieving favorable postoperative outcomes.

## Technical report

The patient was a 12-year-old boy who visited our department with a chief complaint of an aesthetic lip deformity, seeking improvement in both appearance and function. He was diagnosed with a right-sided microform cleft lip through visual inspection and palpation, without genetic testing, and lip reconstruction surgery was planned under general anesthesia. His medical and family history were unremarkable. Clinical findings included a notch in the vermilion and elevation of the Cupid’s bow apex on the affected side, with minimal deformity of the external nose.

**Figure 1:**
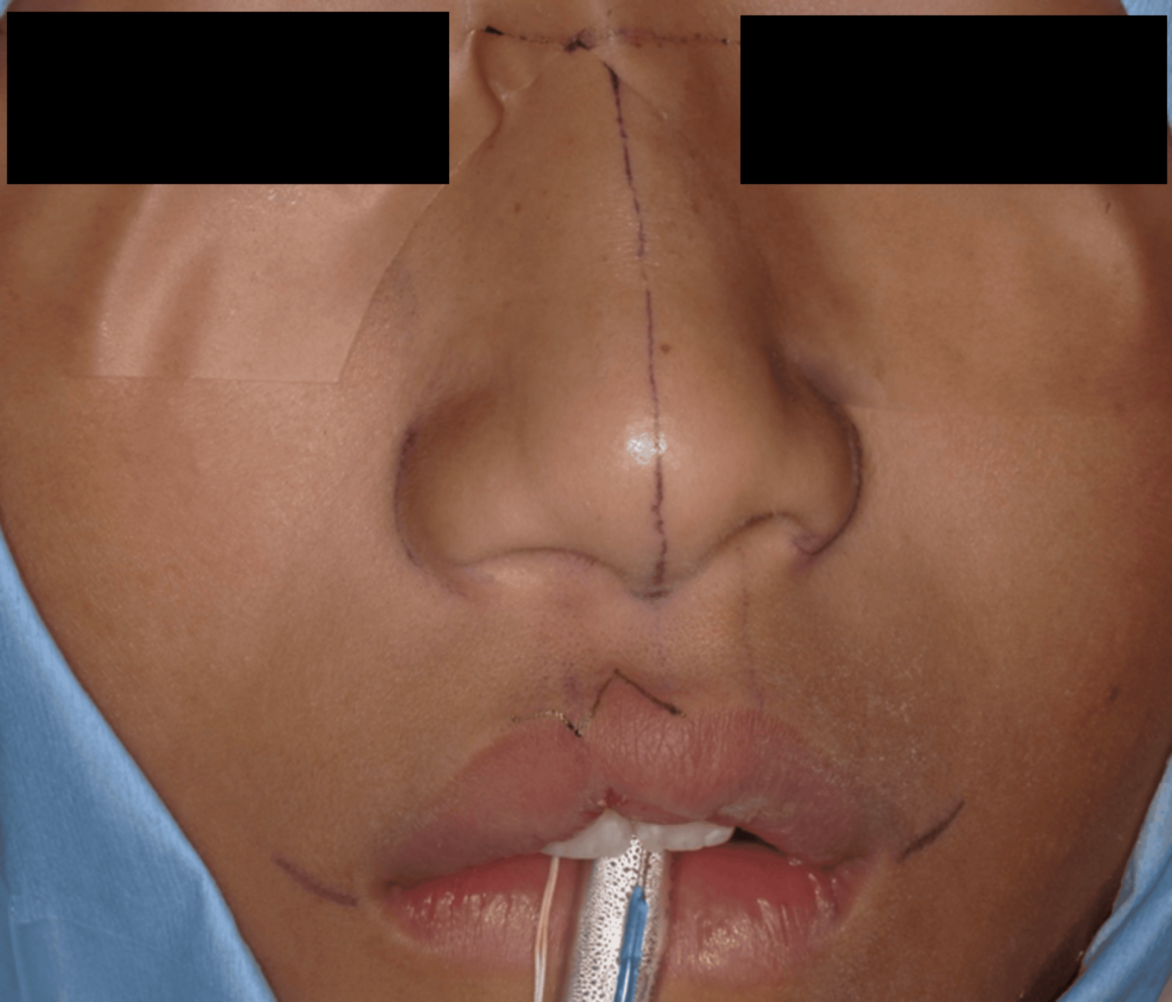
Incision line along the vermilion border

For the surgical procedure, incision lines were first drawn along the vermilion border to minimize scarring and preserve the natural lip contour (Figure 1). A triangular flap was created by extending the white lip into the vermilion and vice versa.

**Figure 2:**
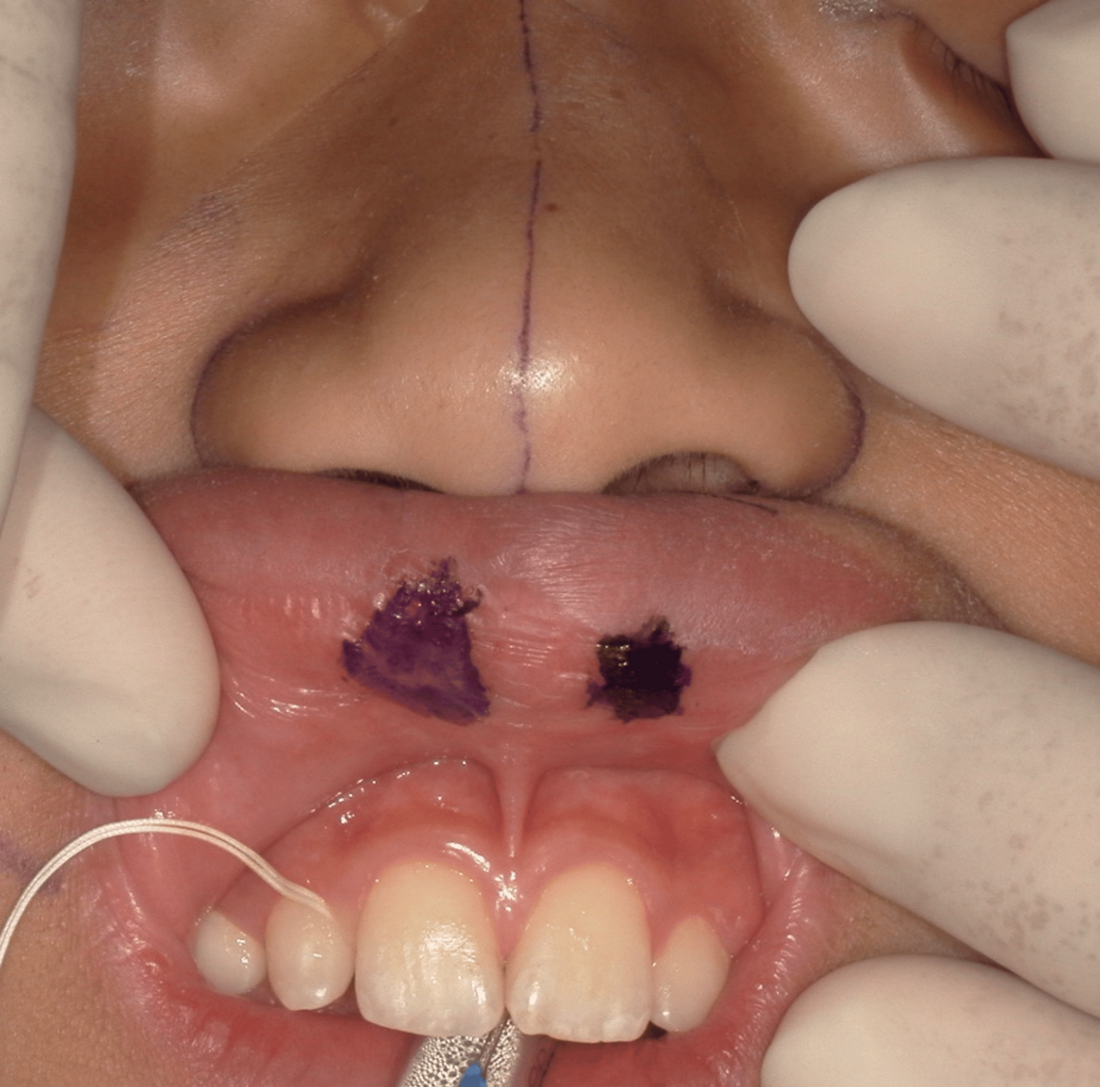
Incision line along the vermilion mucosa The incision line was carefully designed to follow the contour of the depression. The planned area was then incised and excised accordingly.

Next, the extent of the incision was determined to allow for a muscle ring to be formed via a vermilion incision at the site of the orbicularis oris muscle discontinuity (Figure 2).

The skin was incised along the designed lines using a No. 11 scalpel, and a triangular flap was formed to facilitate the reconstruction of the vermilion border. The vermilion border flap was incised and inserted into the corresponding area of the vermilion border. To adjust the length of the vermilion border flap, the incision line was extended, aligning with the Cupid’s bow apex. Excess skin was excised to maintain the lip’s contour, and the lip thickness was adjusted. The vermilion wound was sutured with 7-0 nylon to ensure continuity of the vermilion border.

**Figure 3:**
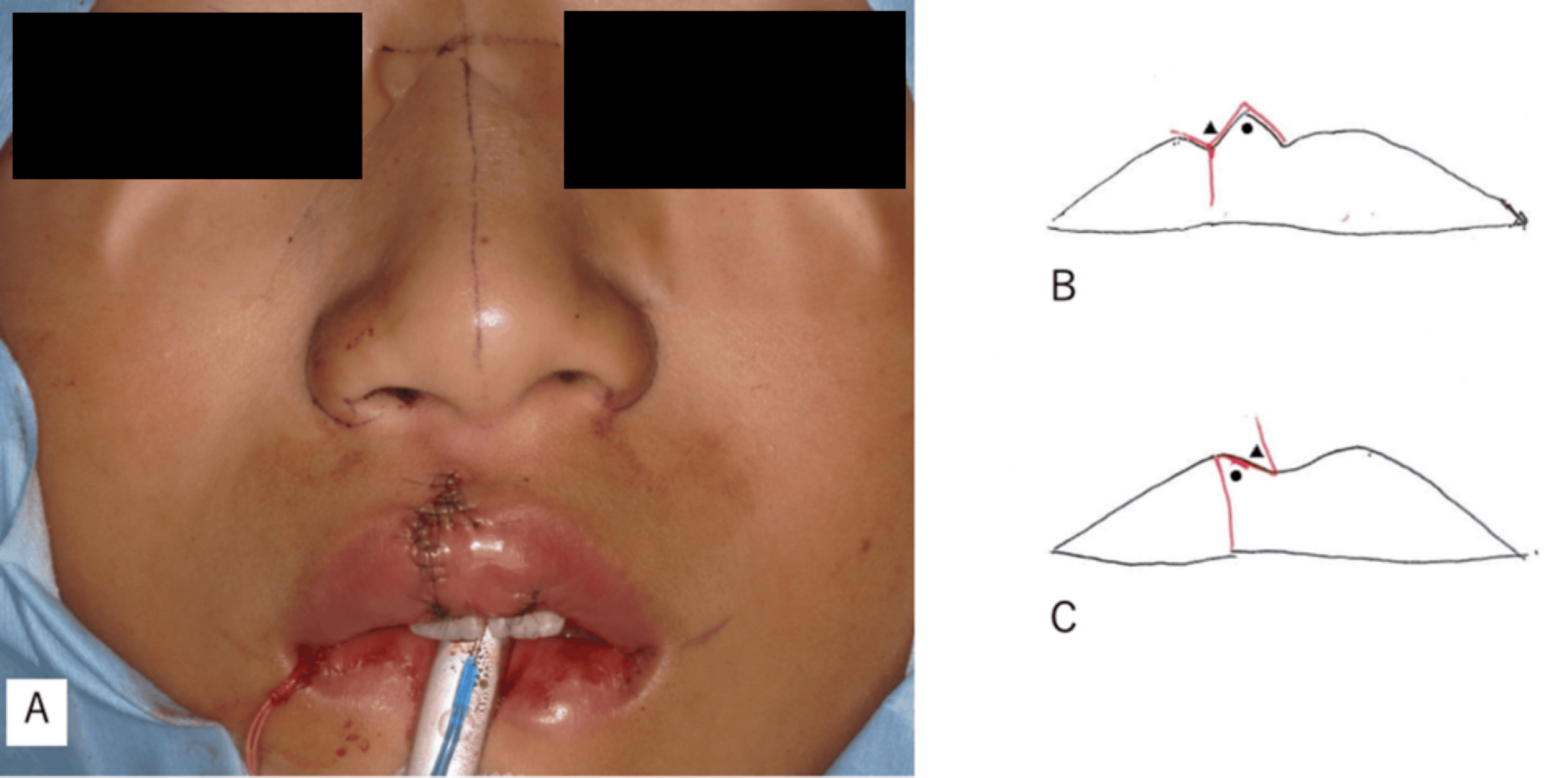
Postoperative outcomes (A) Appearance after surgery. (B) Triangular and circular flaps repositioned beneath the dermis layer. (C) Detachment of the dermis and muscle layers alleviated tissue tension and facilitated vermilion border reconstruction. Circle symbol (●): A red lip flap extending into the white lip. Triangle symbol (▲): A white lip flap extending into the red lip.

**Figure 4:**
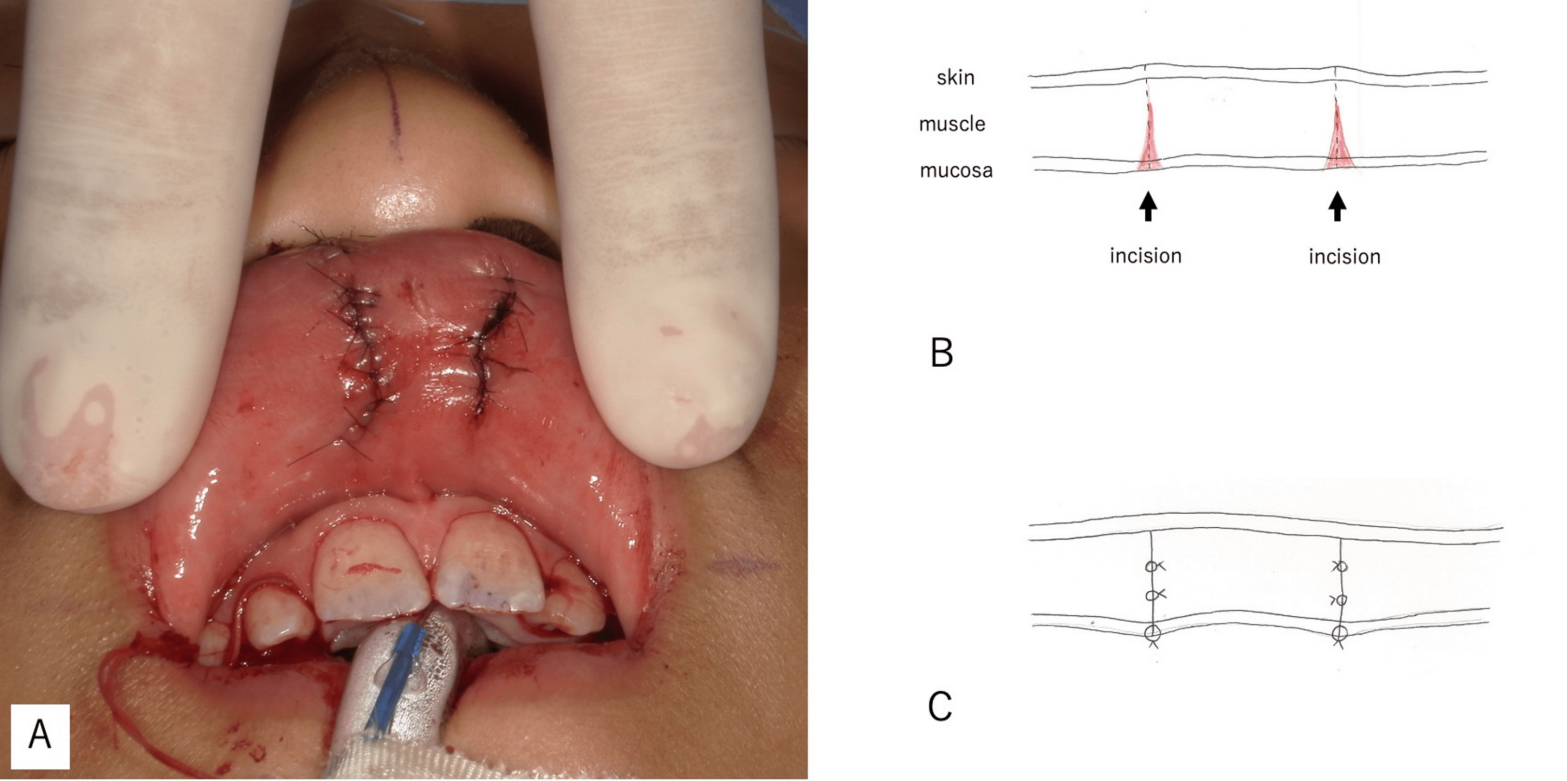
Post-suturing of the vermilion mucosa (A) After suturing the vermilion mucosa. (B) Schematic diagram of the mucosal incision. (C) Schematic diagram illustrating the sutured vermilion mucosa. The depressed area was excised, the muscle layer was dissected beneath the mucosa, and the muscles were overlapped to enhance vermilion volume.

Next, irregularities at the vermilion-cutaneous junction and depressions at the free edge of the labial mucosa were incised. Submucosal dissection was performed, and the discontinuity of the orbicularis oris muscle was overlapped and sutured to maintain thickness. The dermis was sutured with 5-0 polypropylene, and the epidermis was subsequently sutured with 7-0 nylon, completing the procedure (Figure 3, Figure 4).

**Figure 5:**
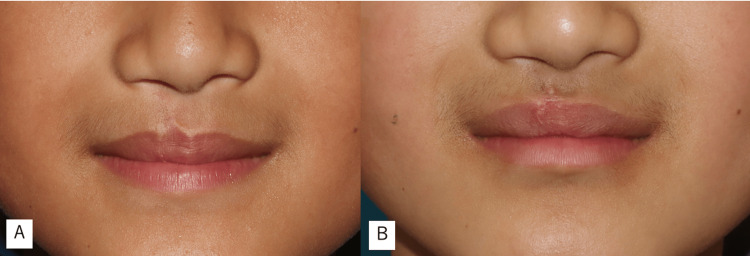
Preoperative and one-year postoperative outcomes (A) Before surgery. (B) One year after surgery. A well-defined Cupid’s bow was successfully achieved.

One year after the operation, both aesthetic and functional outcomes remained favorable (Figure 5).

## Discussion

Microform cleft lip is often not noticeable in early childhood but becomes more prominent as the patient grows, potentially leading to increased self-consciousness. When microform cleft lip presents with differences in vermilion height and discontinuity of the orbicularis oris muscle, surgical techniques such as the rotation-advancement method [6] or the small triangular flap method [7] are commonly used.

However, in cases of minor cleft lips, incisions in the white lip can lead to scarring and disruption of skin texture. Therefore, techniques that minimize incisions in the white lip should be prioritized. For the vermilion, a thorough anatomical evaluation of tissue morphology, including lip prints, is essential to carefully plan the procedure.

Onizuka et al. [1] and Millard [8] have reported that treatment can be successfully performed by tailoring surgical methods to specific symptoms using partial incisions. Histological findings by Lehman and Artz [9] indicate that in microform cleft lip, while the orbicularis oris muscle is partially replaced by collagen fibers, it remains structurally continuous rather than completely severed.

We previously reported that the overlapping technique for the orbicularis oris muscle resulted in a well-formed vermilion in cases of significant tissue defects due to oblique facial clefts and yielded favorable aesthetic outcomes in lip reconstruction following cancer resection. In this case, to address the depression at the free edge of the labial mucosa, we prioritized increasing the thickness of the red lip. Based on our prior experience with the overlapping technique [10,11], we employed it here to enhance muscle continuity and volume. An incision was made to correct the vermilion depression, followed by dissection between the vermilion mucosa and the muscle. Overlapping the muscle created sufficient thickness, successfully improving the depression while avoiding incisions in the white lip. This approach ensured continuity of the orbicularis oris muscle with minimal scarring.

The intraoral incision was aligned with the lip print, and excess tissue was excised. To preserve lip morphology during the procedure, no local anesthetic injections were used; instead, manual compression of the labial artery was employed.

In this case, the surgical approach focused on preserving skin texture and avoiding incisions in the white lip. Continued follow-up is planned to monitor long-term outcomes.

## Conclusions

In this case, microform cleft lip surgery was performed with a minimally invasive approach, preserving the natural skin texture by avoiding incisions in the white lip and focusing on the vermilion border.

The overlapping technique for the orbicularis oris muscle effectively corrected the vermilion depression while maintaining muscle continuity. One year postoperatively, both aesthetic and functional outcomes remained favorable. This approach highlights the importance of precise surgical planning and muscle reconstruction in achieving optimal results. Long-term follow-up will be conducted to evaluate outcome stability and patient satisfaction.
